# Anti-Inflammatory Effects of 81 Chinese Herb Extracts and Their Correlation with the Characteristics of Traditional Chinese Medicine

**DOI:** 10.1155/2014/985176

**Published:** 2014-02-20

**Authors:** Chang-Liang Chen, Dan-Dan Zhang

**Affiliations:** Shanghai University of Traditional Chinese Medicine, Pudong, Shanghai 201203, China

## Abstract

Inducible nitrogen oxide synthase (iNOS) is the primary contributor of the overproduction of nitric oxide and its inhibitors have been actively sought as effective anti-inflammatory agents. In this study, we prepared 70% ethanol extracts from 81 Chinese herbs. These extracts were subsequently evaluated for their effect on nitrogen oxide (NO) production and cell growth in LPS/IFN*γ*-costimulated and unstimulated murine macrophage RAW264.7 cells by Griess reaction and MTT assay. Extracts of *Daphne genkwa* Sieb.et Zucc, *Caesalpinia sappan* L., *Iles pubescens* Hook.et Arn, *Forsythia suspensa* (Thunb.) Vahl, *Zingiber officinale* Rosc, *Inula japonica* Thunb., and *Ligusticum chuanxiong* Hort markedly inhibited NO production (inhibition > 90% at 100 *μ*g/mL). Among active extracts (inhibition > 50% at 100 *μ*g/mL), *Rubia cordifolia* L., *Glycyrrhiza glabra* L., *Iles pubescens* Hook.et Arn, *Nigella glandulifera* Freyn et Sint, *Pueraria lobata* (Willd.) Ohwi, and *Scutellaria barbata* D. Don displayed no cytotoxicity to unstimulated RAW246.7 cells while increasing the growth of LPS/IFN*γ*-costimulated cells. By analyzing the correlation between their activities and their Traditional Chinese Medicine (TCM) characteristics, herbs with pungent flavor displayed potent anti-inflammatory capability. Our study provides a series of potential anti-inflammatory herbs and suggests that herbs with pungent flavor are candidates of effective anti-inflammatory agents.

## 1. Introduction

Inflammation is a self-protection mechanism aiming at removing harmful stimuli, including damaged cells, irritants, or pathogens, and beginning the wound repair process. However, inflammation sometimes induces further inflammation, leading to self-perpetuating chronic inflammation that can cause severe cellular injury and tissue damage [1]. Chronic inflammation has been linked to a wide variety of diseases such as atherosclerosis [2], Alzheimer's disease [3], diabetes [4], and carcinogenesis [5–7].

Nitric oxide (NO), which is mainly generated by inducible nitric oxide synthase (iNOS) under the inflammatory conditions [8–10], plays a key role in each step of the pathological processes during inflammation [11–14]. Selective inhibitors of iNOS have been shown to be both anti-inflammatory and tissue-protective in various inflammatory animal models [15–17] and are thus regarded as promising agents for treating inflammatory diseases. High expression of iNOS can often be detected in human tumors, supporting the notion that chronic inflammation is actively involved in tumor progression [18–21]. In fact, nonsteroidal anti-inflammatory drugs (NSAIDs), including aspirin [22] and tolfenamic acid [23], are currently used for both cancer prevention and treatment [24].

A variety of natural products have been reported to possess anti-inflammatory and anticancer effects in experimental animal models. For example, curcumin has been shown to inhibit cyclooxygenase 2 (COX2) expression and is actually in clinical use as a chemoprevention agent [25–27]. Because of the promises in curcumin, extensive efforts have also been exerted to identify compounds capable of targeting inflammatory mediators [28–30]. A recent study by Liao et al. investigated the potential association between antioxidation capability and the characteristics of Traditional Chinese Medicine (TCM) in 45 commonly used Chinese herbs, in which antioxidation capability of Chinese herbs was found to be correlated with their flavor characteristics [31]. Their findings are very encouraging because it indicates that effective anti-inflammatory agents may potentially be identified from Chinese herbs based on their TCM characteristics.

In our effort to identify effective anti-inflammatory agents, we prepared 70% ethanol extracts from 81 Chinese herbs and subsequently tested their abilities to suppress NO production in murine macrophage RAW264.7 cells costimulated with LPS and IFN*γ*. Moreover, we also analyzed the correlation between anti-inflammatory capacity and TCM characteristics among these herbs. We conclude that herbs with pungent flavor are the strongest in their anti-inflammatory capability.

## 2. Materials and Methods

### 2.1. Chemicals

IFN*γ* was purchased from EMD Millpore Chemicals (Billerica, MA, USA). Bovine serum albumin (BSA), lipopolysaccharide (LPS, *E. coli* 0111: B4), N-(1-naphthyl)-ethylenediamine dihydrochloride (L-NIL), 3-(4, 5-dimethylthiazol-2-yl)-2, 5-diphenyltetrazolium (MTT), naphthylethylenediamine, sulfanilamide, and sodium bicarbonate were all obtained from Sigma-Aldrich Co (St. Louis, MO, USA). RPMI 1640 and trypsin-EDTA were purchased from Life Technologies (Grand Island, NY, USA). Fetal bovine serum (FBS) was purchased from Hyclone Thermo Fisher Scientific (Waltham, MA, USA).

### 2.2. Preparation of 70% Ethanol Extracts of Chinese Herbs

All herbs were obtained from YANG He Tang and Kangqiao Co (Shanghai, China). All 81 herbs chosen for our study have been reported or suggested to have potential anti-inflammatory activities by either TCM literatures or current pharmacological reports. Botanical identification of these herbs was performed by Shanghai Institute for Food and Drug Control (SIFDC). To prepare ethanol extracts, 100 g of each dried herbs was sliced and extracted with 1 L of 70% ethanol at 80°C for three times. Obtained ethanol extracts were evaporated under reduced pressure at temperature 60°C and stored at −80°C. Extracts were dissolved with DMSO before use.

### 2.3. Measurement of Nitrite Production

RAW264.7 cells were plated in 96-well plates (5 × 10^3^ cells per well) for overnight and then replenished with FBS-free medium for 10 h followed by adding 100 *μ*g/mL herb extracts into each well. Cells were costimulated with 100 ng/mL LPS and 10 U/mL IFN*γ* for 24 h, and media were then collected and analyzed for the amount of nitrite, a stable oxidative metabolite and faithful NO indicator, by the Griess reaction as previously described [32]. To do it, 100 *μ*L of Griess reagent (0.1% naphthyl-ethylenediamine and 1% sulfanilamide in 5% phosphoric acid) was mixed with 100 *μ*L of collected medium in a 96-well plate. Mixture was incubated for 10 min at room temperature and then read at 540 nm. The amount of nitrite was calculated based on a standard curve generated with sodium nitrite. Percent inhibition in NO production was calculated with the formula {[(nitrite with herb extract) − (nitrite without herb extract)]/(nitrite without herb extract)} × 100.

### 2.4. Analysis of Cell Viability

Cell viability was determined by MTT assay as previously described [33]. Briefly, RAW264.7 cells were incubated with MTT (5 mg/mL in phosphate-buffered saline, pH = 7.4) for 4 h. Formed MTT formazan was solubilized with 50 *μ*L of 0.01 M HCl buffer containing 10% SDS and 5% isobutanol. Cell growth was determined by reading plates at 570 nm in a microplate reader. The cell viability of control group is considered as 100%.

### 2.5. Statistical Analysis

The direction and magnitude of correlation between variables was done using analysis of *t*-test. *P* values less than 0.05 were considered statistically significant (*P < 0.05).

## 3. Results 

### 3.1. Effect of Herb Extracts on NO Production and Cell Growth

With the aid of Griess assay, we analyzed ethanol extracts of 81 herbs for their anti-inflammatory activity. A wide range of inhibition in NO production was observed with these extracts (Table 1). Extracts of 7 herbs [*Daphne genkwa* Sieb.et Zucc, *Caesalpinia sappan* L.,   *Iles pubescens* Hook.et Arn, *Forsythia suspensa* (Thunb.) Vahl, *Zingiber officinale* Rosc, *Inula japonica* Thunb., and *Ligusticum chuanxiong* Hort] blocked over 90% NO production in LPS/IFN*γ*-stimulated RAW264.7 cells (Table 1). Among the extracts that elicited over 50% inhibition in NO production, *Rubia cordifolia*  L., *Glycyrrhiza glabra*  L., *Iles pubescens* Hook.et Arn, *Nigella glandulifera* Freyn et Sint, *Pueraria lobata* (Willd.) Ohwi, and *Scutellaria barbata* D. Don showed no cytotoxicity to unstimulated RAW264.7 cells while significantly increased the viability of LPS/IFN*γ*-stimulated cells (Table 1). However, *Daphne genkwa* Sieb.et Zucc, which has the strongest inhibitory effect on NO production, was moderately toxic to RAW264.7 cells (Table 1).

### 3.2. Correlation between Anti-Inflammatory Potency and TCM Characteristics of Herbs

Analyzing the TCM characteristics of 10 herbs that display the strongest inhibitory effect on NO production in LPS/IFN*γ*-stimulated RAW264.7 cells, we found that most of them are in the categories of bitter or pungent flavor, warm nature, and lung or liver meridian distributions (Table 2). To correlate the TCM characteristics to anti-inflammatory effect in these herbs, we categorized TCM characteristics of these herbs that were able to abolish 50% of NO production in LPS/IFN*γ*-stimulated RAW264.7 cells.  Table 3 showed that herbs with greater anti-inflammatory effect were distributed in a significantly higher percentage in those characterized as bitter/pungent flavors, warm nature, and liver/lung meridian distributions. These results suggest that anti-inflammatory herbs may possess common characteristics that are of pungent/bitter flavor, warm nature, and lung/liver meridian.

### 3.3. Correlation between Cell Protective Effect and TCM Characteristics of Herbs

**Figure 1:**
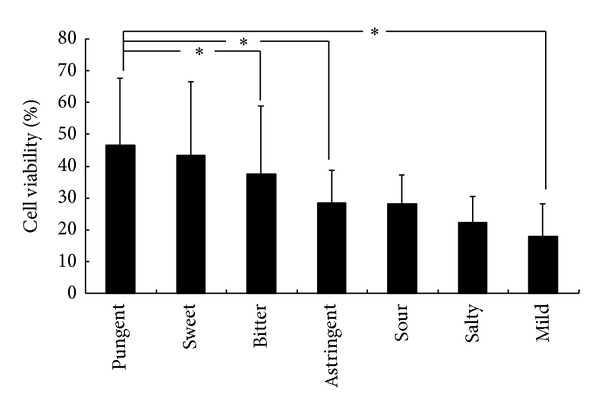
Comparison of herbs with cell viability in different flavors. Average of cell viability of LPS/IFN*γ*-stimulated RAW264.7 cells treated with herbs belonging to different flavor. *P < 0.05.

Chronic inflammation often leads to cell damage and thus agents capable of deterring this process are actively sought. Examining 21 herbs with the TCM characteristic of pungent flavor, we observed that, under the costimulation of LPS and IFN*γ*, RAW264.7 cells treated with these herb extracts displayed 90% increase in cell viability (Table 4). Moreover, herbs with pungent flavor also conferred the highest degree of cell protection in LPS/IFN*γ*-stimulated cells in comparison with herbs with other flavors (Figure 1).

## 4. Discussion

Overproduction of NO due to the elevated iNOS expression has been convincingly linked to the pathogenesis of chronic inflammation and cancer [34]. Hence, agents that can selectively suppress iNOS-generated NO production should be effective to treat chronic inflammation and to prevent cancer. In fact, recent studies demonstrate that selective iNOS inhibitors L-NIL and 1400 W are therapeutically effective as anti-inflammation and anticancer drugs [35, 36].

Macrophages play a critical role in regulating inflammation. Macrophages are activated by external stimuli and activated macrophages produce various inflammatory mediators such as NO and reactive oxygen species. Chinese herbs are the rich sources for anti-inflammatory agents and efforts have been made to identify effective components in these herbs [37, 38]. Taking advantage of the well-established RAW264.7 cell model, we evaluated 81 herb extracts for their inhibitory effect on LPS/IFN*γ*-induced NO production. Among them, the extract of *Daphne genkwa* Sieb.et Zucc showed the strongest inhibitory effect on NO production. The constituents isolated from *Daphne genkwa* Sieb.et Zucc were previously reported to provoke cytotoxic effect to various tumor cell lines and to suppress outgrowth of transplanted mouse sarcoma S180 in mice [39]. We speculate that anticancer effect of *Daphne genkwa* Sieb.et Zucc may be functionally associated with its anti-inflammatory capability. In our study, we found that *Rubia cordifolia* L. and several others decrease LPS/IFN*γ*-induced NO production without causing significant cytotoxicity to RAW264.7 cells. These herbs may thus be promising candidates as effective drugs to control inflammation and cancer. Although it is currently unclear how these extracts block LPS/IFN-induced NO production in RAW264.7 cells, the finding that Mollugin suppresses the inflammatory response by blocking the Janus kinase-signal transducers and activators of transcription signaling pathway [40] implicates that herbs may target the different steps of the signaling cascade mediating LPS/IFN-induced NO production to exert their anti-inflammatory roles.

Based on the theory of TCM, we classified these 81 herbs according to distinct flavors (pungent, sweet, sour, bitter, astringent, salty, or mild), natures (cold, cool, moderate, warm, or hot), and meridian distributions (liver, kidney, heart, spleen, etc.). Our study showed that the TCM characteristic of flavor correlated very well with the potency to inhibit NO production—pungent flavor is the strongest, bitter is slightly weaker than pungent, sweet flavors is intermediate, and astringent, salty, mild, or sour flavor is weak or not effective. TCM characteristics of nature and meridian distribution are also associated with the potency to inhibit NO production. For instance, higher percentage of herbs with the capability to block NO production has the characteristics of warm nature. Characteristics of liver and lung meridians are the major meridian distributions found in herbs whose extracts can block 50% of NO production. Taken together, we reason that TCM characteristics can potentially be very useful to guide the search for effective anti-inflammatory agents in Chinese herbs.

TCM characteristic is a systematic expression of the distinct property elicited by Materia Medica in humans. Theory of flavors in TCM constitutes the core context of Chinese herb usage guidance. In TCM, the characteristic of flavor is the combination of both real taste and curative effect. According to Shen Nong Ben Cao Jing (Shennong's Classic of Material Medica), an important TCM book firstly written on Chinese herbal flavor and property theory, pungent flavor, which is related to lung meridian, can disperse the internal heat with sudorifics which in turn promote the circulation of Qi and blood. Herbs with pungent flavor have actually been used for thousand years in China to invigorate the circulation of blood and break the block of Qi. The fact that inflammation-related diseases are associated with the symptom of Qi and blood blockage may explain the effectiveness of herbs with pungent flavor to suppress inflammation.

Our study was limited to the investigation of 81 herb extracts on their effect on LPS/IFN*γ*-induced NO production and cell growth in macrophage RAW264.7 cells. The results generated from this study nevertheless support a close association between modern pharmacology/biomedical science and TCM theory. TCM theory was developed based on thousand years of clinical experience, and the material and pharmacological basis of TCM remains to be explained by the modern biomedical science. We believe that this study has contributed toward this goal.

## Figures and Tables

**Table 1:** Effect of herb extracts on NO production and cell viability in simulated and resting RAW264.7 cells.

Plant name and authority	Part used^a^	Stimulation cells		Resting cells		Yield^f^
		Percent inhibition of NO^b^	Cell proliferation (%)^c^	NO production (*μ*M)^d^	Cytotoxicity (%)^e^	
*Acanthopanax senticosus* (Rupr.et Maxim.) Harms	SR	74.66 ± 0.01	97.64 ± 0.09	4.28 ± 0.01	90.76 ± 0.04	5.55
*Acanthopanax gracilistylus* W. W. Smith	BK	12.11 ± 0.01	66.29 ± 0.01	1.00 ± 0.01	99.16 ± 0.01	23.75
*Achyranthes bidentata* Bl.	RT	−11.62 ± 0.02	68.78 ± 0001	4.13 ± 0.03	73.25 ± 0.03	31.25
*Acorus tatarinowii *Schott.	SR	11.82 ± 0.01	47.40 ± 0.09	0.92 ± 0.01	98.57 ± 0.01	17.75
*Actinidia arguta* (Sieb.et Zucc.) Planch.ex Miq.	RT	54.67 ± 0.02	40.79 ± 0.03	1.18 ± 0.01	103.69 ± 0.01	7.42
*Actinidia valvata *Dunn	RT	32.11 ± 0.01	51.09 ± 0.01	2.94 ± 0.01	22.07 ± 0.01	7.36
*Alisma orientalis *(Sam.) Juzep.	ST	46.63 ± 0.04	53.01 ± 0.02	3.86 ± 0.02	101.11 ± 0.04	5.69
*Allium macrostemon* Bge.	ST	25.43 ± 0.03	73.53 ± 0.04	2.11 ± 0.01	93.74 ± 0.02	38.87
*Aloe barbadensis* Miller	LF	15.31 ± 0.10	35.02 ± 0.02	3.86 ± 0.02	94.60 ± 0.04	10.71
*Amomum villosum *Lour.	FR	35.56 ± 0.02	61.15 ± 0.04	4.57 ± 0.01	100.19 ± 0.03	5.18
*Artemisia annua *L.	HR	12.03 ± 0.03	29.48 ± 0.03	1.34 ± 0.01	105.08 ± 0.01	13.62
*Artemisia anomala* S. Moore	HR	59.56 ± 0.05	74.25 ± 0.01	1.61 ± 0.01	102.92 ± 0.02	11.04
*Artemisia capillaris* Thunb.	HR	41.2 ± 0.03	64.62 ± 0.03	4.02 ± 0.01	104.40 ± 0.04	17.36
*Astragalus membranaceus *(Fisch.) Bge.	RT	13.96 ± 0.01	53.76 ± 0.03	4.30 ± 0.01	93.30 ± 0.05	47.06
*Bambusa tuldoides* Munro.	ST	27.32 ± 0.01	75.23 ± 0.01	3.19 ± 0.01	110.67 ± 0.03	1.14
*Bletilla striata* (Thunb.) Reichb. f.	ST	77.52 ± 0.01	95.17 ± 0.08	4.29 ± 0.01	14.37 ± 0.02	18.63
*Caesalpinia sappan* L.	HW	94.27 ± 0.01	103.70 ± 0.01	3.75 ± 0.01	30.92 ± 0.01	10.66
*Carpesium abrotanoides* Linn.	HR	74.85 ± 0.03	53.24 ± 0.02	2.85 ± 0.01	102.95 ± 0.02	11.52
*Carthamus tinctorius *L.	FL	38.89 ± 0.02	89.78 ± 0.02	4.22 ± 0.01	104.26 ± 0.04	45.76
*Celastrus orbiculatus *Thunb.	RT	−6.06 ± 0.01	55.62 ± 0.01	1.30 ± 0.01	101.88 ± 0.03	2.78
*Cinnamomum cassia *Presl.	TW	38.43 ± 0.02	86.03 ± 0.03	4.38 ± 0.01	107.18 ± 0.06	9.32
*Cinnamomum cassia* Presl.	BK	68.31 ± 0.02	97.01 ± 0.04	4.54 ± 0.01	60.18 ± 0.04	11.16
*Curcuma longa* L.	ST	89.32 ± 0.02	108.09 ± 0.05	4.61 ± 0.01	51.43 ± 0.02	9.36
*Codonopsis pilosula *(Franch.) Nannf.	RT	−13.92 ± 0.01	60.45 ± 0.01	1.42 ± 0.01	101.83 ± 0.02	36.04
*Corydalis yanhusuo * W. T. Wang	ST	7.36 ± 0.01	25.78 ± 0.01	0.74 ± 0.01	95.26 ± 0.01	11.14
*Chrysanthemum indicum *L.	FL	−2.87 ± 0.01	97.74 ± 0.01	1.65 ± 0.01	101.07 ± 0.02	26.1
*Curculigo orchioides* Gaertn.	ST	8.81 ± 0.01	29.58 ± 0.02	1.25 ± 0.01	106.48 ± 0.02	8.01
*Curcuma wenyujin *Y. H. Chen et C. Ling	RT	−8.31 ± 0.01	45.33 ± 0.01	0.59 ± 0.01	99.01 ± 0.02	9.423
*Curcuma phaeocaulis* Val.	ST	18.87 ± 0.03	43.53 ± 0.02	3.10 ± 0.01	98.00 ± 0.05	46.14
*Dalbergia odorifera * T. Chen	HW	77.38 ± 0.04	88.27 ± 0.07	1.93 ± 0.01	86.12 ± 0.02	17.6
*Daphne genkwa* Sieb.et Zucc.	FL	99.17 ± 0.01	40.83 ± 0.03	4.25 ± 0.01	70.25 ± 0.04	20.55
*Daphne tangutica* Maxim.	BK	76.12 ± 0.01	91.32 ± 0.16	4.56 ± 0.01	85.67 ± 0.11	4.75
*Drynaria fortunei *(Kunze) J. Sm.	ST	6.46 ± 0.04	54.58 ± 0.01	1.15 ± 0.01	99.59 ± 0.02	11.458
*Epimedium brevicornum *Maxim.	LF	−43.84 ± 0.02	135.36 ± 0.02	1.06 ± 0.01	101.69 ± 0.02	13.78
*Euodia rutaecarpa* (Juss.) Benth.	FR	56.35 ± 0.02	23.68 ± 0.01	1.80 ± 0.01	41.84 ± 0.03	33.89
*Forsythia suspensa *(Thunb.) Vahl	FR	91.93 ± 0.01	34.44 ± 0.02	4.24 ± 0.01	27.32 ± 0.01	26.12
*Gardenia jasminoides* Ellis	FR	15.89 ± 0.01	50.48 ± 0.03	4.27 ± 0.01	129.77 ± 0.09	29.8
*Glycyrrhiza glabra *L.	SR	66.62 ± 0.01	107.8 ± 0.07	0.76 ± 0.01	109.65 ± 0.03	18.57
*Iles pubescens *Hook.et Arn.	RT	65.3 ± 0.02	106.52 ± 0.04	4.15 ± 0.01	117.70 ± 0.10	7.09
*Ilex latifolia *Thunb.	LF	32.33 ± 0.09	79.67 ± 0.01	3.42 ± 0.01	54.11 ± 0.04	19.14
*Inula japonica* Thunb.	FL	91.19 ± 0.01	84.48 ± 0.03	0.86 ± 0.01	100.42 ± 0.01	17.7
*Inula linariifolia* Turez.	HR	76.43 ± 0.01	129.93 ± 0.19	4.04 ± 0.01	84.41 ± 0.03	10.91
*Isatis indigotica* Fort.	LF	47.61 ± 0.02	86.83 ± 0.05	1.56 ± 0.01	106.24 ± 0.02	24.43
*Isatis indigotica *Fort.	RT	26.48 ± 0.02	53.51 ± 0.08	1.00 ± 0.01	103.22 ± 0.01	26.78
*Ligusticum chuanxiong* Hort.	SR	91.13 ± 0.01	79.46 ± 0.05	3.88 ± 0.01	81.82 ± 0.04	28.1
*Lonicera japonica* Thunb.	FL	47.87 ± 0.02	86.17 ± 0.04	1.38 ± 0.01	107.12 ± 0.01	39.55
*Magnolia biondii* Pamp.	FL	−15.35 ± 0.01	82.89 ± 0.01	3.27 ± 0.01	102.22 ± 0.03	15.39
*Morus alba* L.	TW	50.78 ± 0.01	72.21 ± 0.06	4.69 ± 0.01	93.67 ± 0.03	7.88
*Nelumbo nucifera* Gaertn.	FR	21.96 ± 0.02	95.84 ± 0.11	4.53 ± 0.04	105.37 ± 0.04	17.55
*Nigella glandulifera *Freyn et Sint.	SD	78.56 ± 0.01	95.88 ± 0.04	2.58 ± 0.01	113.01 ± 0.01	10.05
*Oldenlandia diffusa* (Willd.) Roxb.	HR	43.62 ± 0.02	62.44 ± 0.05	4.12 ± 0.01	69.83 ± 0.02	11.58
*Ophiopogon japonicus * (L.f.) Ker-Gawl.	RT	9.31 ± 0.01	65.68 ± 0.05	0.62 ± 0.01	96.24 ± 0.01	39.34
*Paeonia veitchii *Lynch	RT	61.27 ± 0.05	49.03 ± 0.01	2.95 ± 0.01	101.88 ± 0.03	22.17
*Paeonia lactiflora * Pall.	RT	−8.32 ± 0.01	77.45 ± 0.01	1.16 ± 0.01	98.85 ± 0.03	16.01
*Paeonia suffruticosa* Andr.	BK	31.64 ± 0.04	64.88 ± 0.02	4.15 ± 0.01	70.23 ± 0.01	28.7
*Panax ginseng * C. A. Mey.	SR	26.73 ± 0.04	71.25 ± 0.06	0.92 ± 0.01	101.26 ± 0.01	36.617
*Perilla frutescens* (L.) Britt.	HR	11.22 ± 0.05	64.07 ± 0.02	2.23 ± 0.01	103.16 ± 0.01	12.36
*Peucedanum praeruptorum* Dunn	RT	66.44 ± 0.02	102.58 ± 0.17	4.51 ± 0.01	20.67 ± 0.07	13.07
*Polygonatum odoratum* (Mill.) Druce	ST	−3.64 ± 0.02	47.88 ± 0.01	1.00 ± 0.01	97.64 ± 0.01	32.28
*Polygonum multiflorum* Thunb.	RT	36.49 ± 0.02	63.84 ± 0.02	4.91 ± 0.01	73.7 ± 0.02	12.57
*Poria cocos* (Schw.) Wolf	SC	56.75 ± 0.04	12.61 ± 0.06	1.34 ± 0.01	49.13 ± 0.03	2.21
*Psoralea corylifolia* L.	FR	41.35 ± 0.04	93.39 ± 0.04	1.16 ± 0.01	7.93 ± 0.01	5.34
*Pueraria lobata *(Willd.) Ohwi	RT	58.64 ± 0.03	93.10 ± 0.08	0.68 ± 0.01	101.30 ± 0.02	20.25
*Pyrola calliantha *H. Andres.	HR	20.09 ± 0.07	50.68 ± 0.02	3.04 ± 0.01	106.48 ± 0.04	11.6
*Rehmannia glutinosa* Libosch.	RT	−14.78 ± 0.01	38.15 ± 0.01	0.45 ± 0.01	96.41 ± 0.01	39.67
*Rosa laevigata* Michx.	FR	29.37 ± 0.02	69.39 ± 0.03	4.40 ± 0.01	91.48 ± 0.06	22.8
*Rubia cordifolia *L.	SR	69.99 ± 0.03	113.22 ± 0.12	5.30 ± 0.01	102.03 ± 0.06	12.67
*Salvia miltiorrhiza* Bge.	SR	7.35 ± 0.01	82.25 ± 0.14	2.02 ± 0.01	100.35 ± 0.01	40.42
*Santalum album* L.	HW	36.59 ± 0.02	61.80 ± 0.03	4.61 ± 0.01	63.65 ± 0.16	7.25
*Saposhnikovia divaricata *(Turcz.) Schischk.	RT	6.73 ± 0.01	56.66 ± 0.01	3.02 ± 0.01	92.08 ± 0.10	20.51
*Scutellaria baicalensis *Georgi	RT	23.55 ± 0.01	69.68 ± 0.01	3.07 ± 0.01	100.93 ± 0.01	47.06
*Scutellaria barbata *D. Don	HR	53.51 ± 0.03	98.59 ± 0.03	4.28 ± 0.01	101.75 ± 0.04	21.39
*Satsstrea japonica *(Thunb.) De.	BK	70.55 ± 0.01	126.05 ± 0.14	4.19 ± 0.01	91.61 ± 0.03	4.66
*Spatholobus suberectus *Dunn.	ST	33.79 ± 0.01	27.24 ± 0.01	4.98 ± 0.01	92.21 ± 0.07	16.07
*Stephania tetrandra* S. Moore	RT	52.29 ± 0.06	8.38 ± 0.01	2.06 ± 0.01	98.80 ± 0.03	11.03
*Tribulus terrestris* L.	FR	73.48 ± 0.02	71.80 ± 0.09	4.15 ± 0.01	87.66 ± 0.05	8.44
*Trichosanthes kirilowii *Maxim.	PE	−2.38 ± 0.03	29.54 ± 0.01	1.07 ± 0.01	101.00 ± 0.01	35.97
*Typha angustifolia* L.	PL	78.99 ± 0.05	48.80 ± 0.01	3.66 ± 0.01	85.85 ± 0.02	7.09
*Typhonium giganteum *Engl.	ST	7.41 ± 0.01	47.14 ± 0.01	0.46 ± 0.01	94.68 ± 0.02	24.56
*Xanthium sibiricum* Patr.	HR	76.34 ± 0.04	94.41 ± 0.07	4.46 ± 0.01	83.82 ± 0.06	5.73
*Zingiber officinale* Rosc.	SR	91.28 ± 0.01	98.31 ± 0.05	2.20 ± 0.01	41.37 ± 0.04	10.10
L-NIL^g^		35.2 ± 0.01	84.29 ± 0.01	3.22 ± 0.01	99.95 ± 0.03	

^a^HR: herb; RT: root; ST: stem; LF: leaf; TW: twig; FL: flower; FR: fruit; SD: seed; SC: sclerotium; HW: heartwood; SR: stem and root; PE: pericarp.

^b^Percent inhibition of NO production: Griess reaction was carried out to measure the production of nitrite in LPS/IFN*γ*-stimulated RAW264.7 cells in the absence or presence of 100 *μ*g/mL herb extracts.

^c^Cell growth: MTT was performed to measure cell growth. The growth rate of control (no herb extract treatment) was considered as 100%.

^d^NO production: Griess reaction was used to measure the amount of nitrite in unstimulated RAW264.7 cells in the absence and presence of 100 *μ*g/mL herb extracts.

^e^Cell cytotoxicity: MTT assay was performed to determine cell cytotoxicity of unstimulated RAW264.7 cells treated with herb extracts. Untreated group was considered as 100%.

^f^Percent yield of extract obtained from 70% ethanol extraction of each 100 g dry herb.

^g^Percent inhibition of iNOS activity at the test concentration of 50 *μ*M.

**Table 2:** Characteristics (flavor, nature, and meridian distributions) of the 10 most potent anti-inflammatory herbs.

Plant name and authority	Flavors^a,b^	Natures^a,b^	Meridian distributions^a,b^
*Daphne genkwa* Sieb.et Zucc.	Bitter, pungent	Warm	Lung, spleen, kidney
*Caesalpinia sappan* L.	Sweat, salty	Moderate	Heart, liver, spleen
*Forsythia suspensa* (Thunb.) Vahl	Bitter	Litter cold	Lung, heart, intestinum tenue
*Zingiber officinale* Rosc.	Pungent	Hot	Spleen, stomach, kidney, heart, lung
*Inula japonica* Thunb.	Bitter, pungent, salty	Little warm	Lung, spleen, stomach, intestinum crassum
*Ligusticum chuanxiong* Hort.	Pungent	Warm	Liver, gallbladder, pericardium meridian
*Curcuma longa* L.	Pungent, bitter	Warm	Spleen, liver
*Typha angustifolia* L.	Sweat	Moderate	Liver, pericardium meridian
*Nigella glandulifera *Freyn et Sint.	Sweat, pungent	Warm	Liver, kidney
*Bletilla striata* (Thunb.) Reichb.f.	Bitter, sweet, astringent	Little cold	Lung, liver, stomach

^a^Based on Chinese Pharmacopoeia (2010).

^b^Based on Chinese Materia Medica (1998).

**Table 3:** Percentage distribution of herbs with the ability to inhibit over 50% NO production in each TCM characteristics.

TCM characteristic	Hit extracts (inhibition over 50%)	Percentage of effective herbs (32)	Herbs sharing same flavors	Percentage (in 81 herbs)
Four properties				
Cold	9	28.13	30	37.04
Cool	1	3.13	2	2.47
** **Warm	**11**	**34.38**	33	40.74
Hot	3	9.38	4	4.94
Moderate	8	25	12	14.81
Five flavors				
** **Pungent	**20**	**62.5**	42	51.85
Sweet	9	28.13	30	37.04
** **Bitter	**20**	**62.5**	47	58.02
Sour	0	0	3	3.70
Astringent	2	6.25	6	7.41
Salty	3	9.38	3	3.70
Mild	2	6.25	3	3.70
Meridian distributions				
** **Liver	**18**	**56.25**	43	53.09
** **Lung	**17**	**53.13**	35	43.21
Spleen	13	40.63	29	35.80
Heart	10	31.25	30	37.04
Kidney	8	25	25	30.86
Stomach	7	21.88	22	27.16
Intestinum crassum	4	12.5	9	11.11
Urinary bladder	2	6.25	7	8.64
Gallbladder	2	6.25	6	7.41
Intestinum tenue	1	3.13	2	2.47

**Table 4:** Percentage distribution of herbs with cell protective capability in each TCM characteristics.

TCM characteristics	Hit herbs^a^	Percentage (21 herbs)	Hit herbs^b^	Percentage (43 herbs)
Four natures				
** **Cold	5	23.81	**19**	44.19
Cool	1	4.76	1	2.33
Moderate	5	23.81	5	11.63
** **Warm	**8**	38.10	**15**	34.88
Hot	2	9.52	1	2.33
Five flavors				
** **Pungent	**15**	71.43	**21**	48.84
Sweet	9	42.86	14	32.56
** **Bitter	**13**	61.90	**22**	51.16
Sour	0	0	0	0
Astringent	2	9.52	2	4.65
Salty	2	9.52	1	2.33
Mild	0	0	2	4.651
Meridian distributions				
** **Liver	**9**	42.86	**20**	46.51
** **Lung	**11**	52.38	**18**	41.86
** **Spleen	**10**	47.62	12	27.91
Heart	8	38.10	14	32.56
Kidney	8	38.10	12	27.91
Stomach	4	19.05	13	30.23
Intestinum crassum	3	14.29	3	6.977
Urinary bladder	1	4.76	6	13.95
Gallbladder	0	0	5	11.63
Intestinum tenue	0	0	1	2.326

^a^Herbs with over 90% cell protective capability in stimulated RAW264.7 cells.

^b^Herbs with ability to increase over 90% cell proliferation in resting RAW264.7 cells.
